# Nanoscale Imaging of Whole Cells Using a Liquid Enclosure and a Scanning Transmission Electron Microscope

**DOI:** 10.1371/journal.pone.0008214

**Published:** 2009-12-14

**Authors:** Diana B. Peckys, Gabriel M. Veith, David C. Joy, Niels de Jonge

**Affiliations:** 1 Center for Environmental Biotechnology, University of Tennessee, Knoxville, Tennessee, United States of America; 2 Oak Ridge National Laboratory, Materials Science and Technology Division, Oak Ridge, Tennessee, United States of America; 3 Department of Materials Science and Engineering, University of Tennessee, Knoxville, Tennessee, United States of America; 4 Department of Molecular Physiology and Biophysics, Vanderbilt University Medical Center, Nashville, Tennessee, United States of America; Clarkson University, United States of America

## Abstract

Nanoscale imaging techniques are needed to investigate cellular function at the level of individual proteins and to study the interaction of nanomaterials with biological systems. We imaged whole fixed cells in liquid state with a scanning transmission electron microscope (STEM) using a micrometer-sized liquid enclosure with electron transparent windows providing a wet specimen environment. Wet-STEM images were obtained of fixed *E. coli* bacteria labeled with gold nanoparticles attached to surface membrane proteins. Mammalian cells (COS7) were incubated with gold-tagged epidermal growth factor and fixed. STEM imaging of these cells resulted in a resolution of 3 nm for the gold nanoparticles. The wet-STEM method has several advantages over conventional imaging techniques. Most important is the capability to image whole fixed cells in a wet environment with nanometer resolution, which can be used, e.g., to map individual protein distributions in/on whole cells. The sample preparation is compatible with that used for fluorescent microscopy on fixed cells for experiments involving nanoparticles. Thirdly, the system is rather simple and involves only minimal new equipment in an electron microscopy (EM) laboratory.

## Introduction

About 80% of all microscopy investigations in the life sciences are carried out with light microscopy. Since the introduction of sub-diffraction-limit techniques, so-called nanoscopy techniques, the light microscope has become an even more powerful tool for biologists. The spatial resolution is about 50 nm [1], although values up to 10 nm have been reported for extended image acquisition times [2]. But, when it comes to scientific questions dealing with individual protein localizations in cells the technique of choice is usually transmission electron microscopy (TEM) on account of the size of most proteins in the range of 1–10 nm [3]. Organelles, membranes, and protein complexes are traditionally imaged in thin sections [4]. The cells are fixed, metal-stained, embedded in plastic, and sectioned. Distributions of individual proteins can be investigated using labeling techniques, such as immunogold labeling [5]. Preservation of the native structure can be enhanced by using cryo EM [6], [7]. However, standard EM techniques are not compatible with whole cell imaging and require elaborated specimen preparation (preparation of thin sections), or are limited to the cell edges where the thickness is only a few hundreds of nanometers [8]. Ever since the invention of the electron microscope scientists have attempted to image whole cells in their native liquid state with EM [9], just as in light microscopy. During the past decade advances in materials for electron transparent windows led to useful imaging systems [10], [11]. We have recently demonstrated 4 nm resolution on gold labeled epidermal growth factor (EGF) receptors in whole fixed eukaryotic cells (COS7 cell line) in water [12]. That experiment involved an advanced specimen holder capable of flowing liquid to and from the specimen in the vacuum interior of the electron microscope. Flow is needed to ensure a complete filling of sample compartment with liquid and to exchange the liquid for imaging dynamic events in future experiments. Various biological experiments, however, merely require the recording of high-resolution images of fixed cells. In a wet environment, i.e. an environment containing both water and water vapor, the preservation state of the structure of fixed cells is similar to its living state [13]. The liquid flow can then be omitted and a much simpler (and cheaper) system can be used.

**Figure 1:**
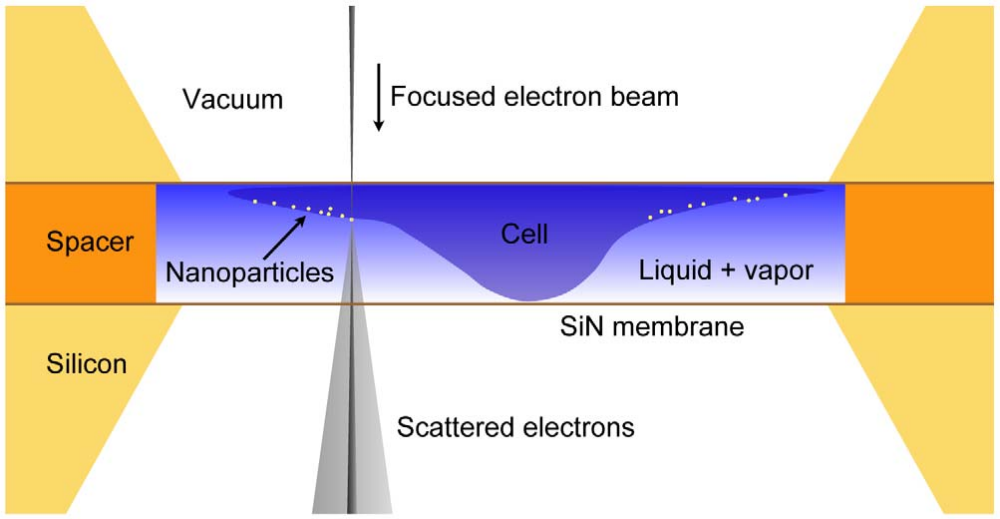
Schematic of the liquid enclosure for wet scanning transmission electron microscopy (STEM). A cell in a wet environment consisting of liquid and vapor is enclosed between two electron-transparent silicon nitride windows. The liquid enclosure is placed in the vacuum of the electron microscope. Images are obtained by scanning the electron beam and detecting elastically scattered transmitted electrons. Labels of a material of a high atomic number can be distinguished.

Here, we present a liquid enclosure (a micro-environmental chamber) for maintaining a wet environment, which can be used for the nanoscale imaging of labeled proteins in/on fixed cells in liquid state. We demonstrate the use of wet STEM on two different samples, whole E. coli bacteria with surface gold labels, and mammalian cells (COS7) incubated for 5 minutes with gold-tagged EGF. Figure 1 shows a schematic drawing of the liquid enclosure formed by two silicon microchips, in which the biological specimen, e.g., bacterial-, or mammalian cells, are placed in aqueous solution. The liquid enclosure has two ultra-thin electron-transparent windows of silicon nitride. The silicon microchips are separated by a spacer and sealed at their sides with epoxy. This liquid enclosure is placed in the vacuum of the electron microscope and a focused electron beam is scanned over the sample. The annular dark field (ADF) detector located below the sample is used to detect electrons that are elastically scattered from the main electron beam. The ADF detector is sensitive to the atomic number of the atoms in the specimen, so-called Z-contrast [14]. It is thus possible to image nanoparticles with a high electron density (high atomic number), that can be used to tag individual proteins, inside a thick (up to about ten micrometer) layer of material of low atomic number, such as water or protein [15].

## Methods

### Gold Labeling of *E. coli* Bacteria

Gold nanoparticles were bound to the amino groups of surface proteins of the E. coli outer membrane. N-succinimidyl 3-(2-pyridyldithio)-propionate (SPDP) (Pierce Biotechnolgies) was used as linker. An E. coli aliquot (BL21DI3) was incubated for 30 minutes with 100 mM SPDP in phosphate buffered saline (PBS). The cells were then fixed with a solution of 2.5% glutaraldehyde (EM grade, SPI) in phosphate buffered saline (PBS), pH 7.4, for 60 minutes. Next, the SPDP was cleaved in 10 mM TCEP Bond Breaker (Pierce Biotechnolgies) stock solution, yielding reactive thiol groups. The solution was then incubated over night with gold nanoparticles (30 nm diameter) (Nanoparts). Last, the bacteria were washed (by centrifugation) several times with PBS, re-suspended in 200 mM NaCl and stored at 4°C until used for imaging. For STEM imaging, a monolayer of E. coli was made by coating the silicon nitride membrane with poly-L lysine and subsequent incubation with the fixed and labeled E. coli for 30 minutes, followed by washing with PBS.

### Cell Culture and Labeling of COS7 Cells with EGF-Gold Nanoparticles

EGF receptors of COS7 cells (African Green Monkey kidney fibroblast) were labeled with gold nanoparticles [12], [16], [17]. Cells were grown in DMEM (ATTC), supplemented with 10% FBS, in a 5% CO_2_ atmosphere, at 37°C. Confluent COS7 cells were harvested using Dulbecco's PBS (ATTC) and CellStripper (Mediatech). For cell attachment the silicon microchips were coated with poly-l-lysine (Sigma Aldrich) at the silicon nitride side. The microchips with the cells were incubated for at least 4 hours or overnight, in a 5% CO_2_ atmosphere, at 37°C. For EGF receptor labeling we used the following procedure. A solution of 10 nm diameter gold-labeled streptavidin (KPL) was diluted in PBS containing 0.5% BSA (PBS-BSA). The gold particles were washed and a 22 nM gold nanoparticle solution in PBS-BSA was incubated with 0.4 µM Biotin-EGF (Invitrogen) for 1 hour at 35°C. Unbound biotin-EGF was removed using a size exclusion column. The filtrate, containing EGF-gold nanoparticles (EGF-Au) was diluted with Tyrode's buffer (CaCl_2_ 1.8 mM, MgCl_2_ 1.0 mM, KCl 2.7 mM, NaHCO_3_ 12.0 mM, NaCl 137 mM, NaH_2_PO_4_ 0.4 mM, D-Glucose 5.5 mM, pH 7.4, Sigma-Aldrich), supplemented with 14.5 mM D-Glucose and 0.5% BSA (Tyrode's-BSA), washed once and re-suspended to yield 10 nM EGF-Au in Tyrode's-BSA.

Four hours prior to EGF-Au labeling, the medium in the wells was exchanged by serum free DMEM. Afterwards, the cells were washed once with Tyrode's-BSA. 11 µL droplets of EGF-gold nanoparticle solution were placed inside the rim of 4 mm diameter plastic wells and 1 silicon microchip per droplet was placed, inclined upside down on the droplet. The microchips were then stored in a closed box with a 100% humidity environment. The microchips remained in this environment for 5 minutes at room temperature, under a 1 Hz wobbling agitation of the box (using a gyratory shaker). The microchips were washed with PBS and fixed for 15 minutes in 4% glutaraldehyde in PBS, pH 7.4, washed 3 times with PBS, once with 10% PBS in water, incubated for 5 min in 100 mM glycine to quench un-reacted aldehyde groups after fixation, washed twice with 10% PBS and left in this solution at 4°C until imaging. Further details of this method and control experiments are described elsewhere [12].

### The Silicon Devices for Wet STEM Imaging

**Figure 2:**
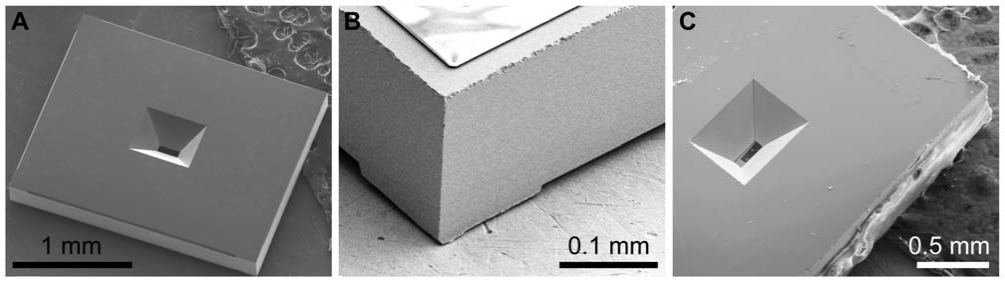
Scanning electron microscopy (SEM) images of the silicon microchips. The SEM images were recorded at 10 kV (S4700 Hitachi). (A) Image of the backside of a microchip showing the opening for the silicon nitride window. (B) Close-up of the diced edge of the microchip. The SU8 spacer layer is visible at the top (the layer charges under the influence of electron beam irradiation). (C) Image of a liquid enclosure assembled from two microchips and closed at all sides with epoxy. The bottom microchip is visible through the silicon nitride window confirming the alignment of the top- and the bottom window.

The key components of the wet STEM system are two silicon microchips supporting silicon nitride windows of 50 nm thickness, which are transparent to the electron beam of the STEM (200 kV in our case) [12], [18]. A silicon microchip is shown in **Figure 2**A. The outer dimensions were 2.00×2.60×0.30 mm^3^. The size of the silicon nitride window was 50×200 µm^2^. This size, thickness, and rectangular shape presented an optimum balance between field of view and strength to withstand this pressure difference occurring when the liquid enclosure is placed in the vacuum of the electron microscope. The thickness of 50 nm was found to be optimal for STEM imaging. Thicker windows caused electron beam blurring, while thinner windows exhibited an increased risk of breaking. The extended length in the other dimension of 200 µm allowed the imaging of multiple cells, which is desirable for biological experiments.

The custom designed microchips were fabricated using low stress silicon nitride of 50 nm thickness deposited with a low-pressure chemical vapor deposition process onto both sides of 300 µm thick silicon wafers. The silicon nitride film on one side of the wafer was patterned using photolithography and reactive ion etching to expose the silicon in locations defining the window areas. The wafers were then placed in a heated KOH bath that etched the exposed silicon (but not silicon nitride), thus forming the windows. The microchips were separated from the wafer by dicing resulting in individual microchips with vertical edges. The manufacturing procedure was optimized such that the edges of the microchips were defined with a precision of ±10 µm with respect to the silicon nitride windows. **Figure 2**B depicts the corner of a microchip.

One set of microchips contained an additional spacer layer to set the height if the sample region between the microchips. The spacer layer covered most of the surface of the microchips and left a specimen region open around the position of the window. The spacer consisted of SU8, an epoxy-based photo resist. Depositing SU8 on the wafer after etching the silicon, and patterning this material using photolithography formed the spacer layer. The SU8 material on the surface of the microchip can be seen at the top in **Figure 2**B. The spacer did not extend until the very edge of the microchips to prevent detachment of the spacer during dicing.

### Wet Sample Assembly for STEM Imaging

**Figure 3:**
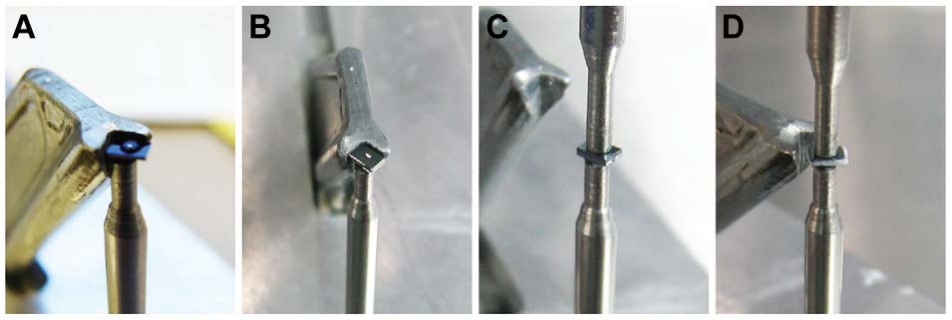
Pictures showing assembly of the liquid enclosure. These pictures were made of a test device without a biological sample. (A) The bottom window with a droplet of liquid is positioned on the pole of the loading device with the silicon nitride side facing up. A retractable aligner of the loading device supports two edges. (B) The top window containing the biological specimen is placed facedown on the bottom microchip. (C) A pole with a weight presses on the stack of windows. (D) The vacuum epoxy (white), serving to glue both microchips together and to vacuum-seal the micro-chamber, is visible at the sides of the micro device.

The liquid enclosure was constructed from two silicon microchips with the help of a simple loading device of local design. The microchip with the wet biological sample was placed with the silicon nitride facing up on the pole of the loading device and supported at two sides by a retractable aligner. Prior to loading, the biological sample was placed in a solution of 50% H_2_O, 50% glycerol and 100 mM NaCl. Glycerol was added in order to increase the viscosity of the liquid, thus preventing rapid evaporation. The salt provided electrical conductance in the liquid to reduce charging effects caused by secondary electrons during STEM imaging. **Figure 3** shows four steps of the loading procedure for a non-biological test sample, in which a dry window was first placed on the pole of the loading device and a droplet of 0.5 µl of the above solution was placed in the middle of the silicon nitride window. The second silicon microchip (with SU8 spacer) was placed face down (etched opening up) on the droplet and both microchips were aligned at their sides using the aligner. The upper pole of the loading device containing a weight (not shown) was lowered to press both microchips together. Pressing the microchips together pushed excess liquid out of the gap between the microchips. The aligner was then retracted and pressing with tweezers on two sides further aligned the microchips. On account of the precisely diced edges, the silicon microchips aligned within ±20 µm using this procedure, and thus the silicon nitride windows overlapped, as needed for STEM imaging. Finally, the microchips were sealed with high-vacuum epoxy (Varian) and dried for a minimum of 2 hours, resulting in a monolithic liquid enclosure. **Figure 2**C shows an assembled liquid enclosure with overlapping windows.

### Verification of the Presence of Water

The existence of water in the liquid enclosures was verified by measuring the infrared absorbance with a BioRad 575C nitrogen purged Fourier Transform Infrared Spectrometer (FTIR) for the bacterial sample, and with a Nexus 670 FTIR, Thermo Nicolet, for the COS7 sample (for reasons of availability of the equipment). Liquid enclosures were mounted on a sample holder designed to focus the infrared beam through the silicon nitride window in the liquid enclosure. Background data was collected on a single silicon nitride window. Data were collected on liquid enclosures after STEM imaging to confirm that water was still in the enclosure after it had been exposed to the vacuum of the microscope from the visibility of the absorbance in the infrared at the characteristic stretching frequency of 3360 cm^−1^ of -OH groups. For comparison a dummy was made in the same way as the real enclosure, but it was not sealed with epoxy. Exposing the dummy enclosure to vacuum led to the removal of the water and the -OH stretching band around 3360 cm^−1^ was absent, thus confirming the absence of water in the dummy enclosure.

Although the presence of water in the micro-environmental chamber was verified, the use of these liquid enclosures does not guarantee the complete filling of the entire volume of the micro-chamber with water (liquid flow is needed for complete filling [12]). In test experiments we have observed the occurrence of bubbles of the size of several tens of micrometers inside the liquid. The occurrence of micro-bubbles may be due to the silicon nitride windows bulging outward into the vacuum (under-pressure at the vacuum side), thus reducing the pressure in the micro-chamber, which may lead to partial de-gassing of the water. Thus, the wet environment in the micro-chamber likely contains water vapor in addition to the liquid water. The environment containing both liquid and vapor can be advantageous for certain samples with respect to a system filled entirely with liquid on account of an effective decrease of the liquid thickness since the resolution is inversely proportional to the square root of the liquid thickness [12].

### STEM Imaging

**Figure 4:**
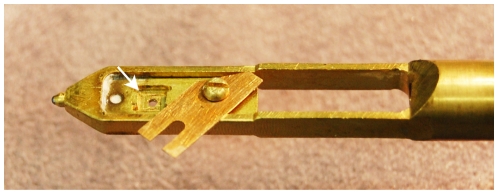
Picture of the tip of the modified specimen holder (Hitachi type). The liquid enclosure fits into the slot indicated by the arrow. A metal blade and a screw serve to fix the liquid enclosure in place.

For STEM imaging we used a 200 kV STEM (Hitachi HD2000) in high-resolution mode with an approximate probe current of 0.1 nA. A modified single-tilt TEM/STEM specimen holder containing a slot fitting the liquid enclosure (**Figure 4**) was used to position the micro-environmental chamber in the STEM. The liquid enclosure was placed up side down in the specimen holder, such that the biological sample was on top of the liquid (the electron beam entered the device from the top). A STEM imaging session started with the adjustment of the vertical position of the stage by focusing on debris on top of the liquid cell using the secondary electron detector positioned above the specimen. The sample was then imaged in transmission mode with the ADF detector. The brightness and contrast settings were adjusted for the large background signal associated with the liquid. Images of a size of 1280×960 pixels were recorded. The imaging time was 10 seconds. The contrast and brightness of the images were adjusted later for maximal visibility of the labels, and the images were cropped (using ImageJ software).

## Results

### Wet STEM Imaging of Gold-Labeled *E. coli* Cells

**Figure 5:**
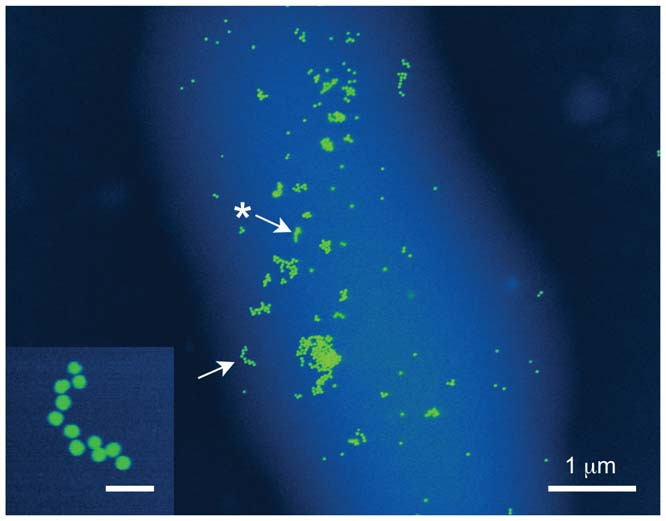
Wet STEM imaging of labeled *E. coli* bacterium with surface gold labels having a diameter of 30 nm. The inset is a selected area of a second image recorded at a 2.3 times larger magnification, showing the particles indicated by the arrow. Scale bar inset 100 nm. The arrow with the star points to gold labels that are out-of-focus. The signal intensity was color-coded, such that gold labels appear in yellow, the cell in light blue and the background in dark blue.

Gold nanoparticles of a diameter of 30 nm were covalently bound to the amino groups of the membrane proteins of *E. coli* bacteria. The labeled *E. coli* bacteria in liquid were loaded in the liquid enclosure and then imaged with a 200 kV STEM. **Figure 5** shows a gold-labeled *E. coli* bacterium displaying gold nanoparticles, which are visible as yellow circles. The STEM contrast obtained on the carbon-based cellular material of the bacterium (the light blue area in the image) is less than that for gold due to the much lower atomic number than gold. The contours of the cell can be recognized as blue shape, intracellular structure is not visible. The left arrow points towards a cluster gold nanoparticles that was also imaged a second time at a higher magnification. The star-labeled arrow points to a position where the image of the gold nanoparticles is blurred. We assume that these nanoparticles with blurred edges were at the bottom of the bacterium, while the nanoparticles with sharp edges were at the top. Blurring primarily occurs because these nanoparticles at different vertical positions are imaged with an out-of-focus electron beam (the focal depth of the STEM used is about 0.1 µm). Secondly, liquid and cellular material in the bacterium leads to scattering of the electron beam, such that labels at the bottom of the bacterium will appear blurred.

### STEM Imaging of Wet Gold-Labeled COS7 Cells

**Figure 6:**
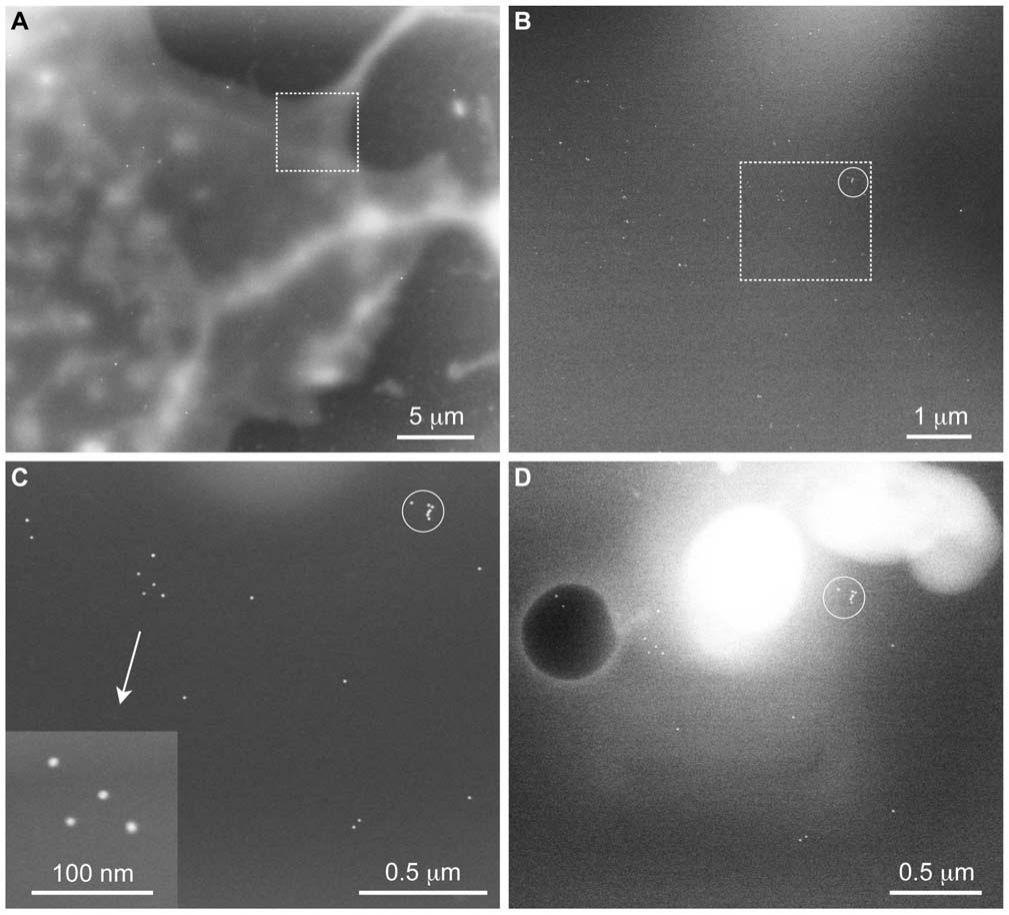
Wet STEM imaging of gold-labeled (10 nm diameter) epidermal growth factor receptors in COS7 cells. (A) Image of a part of a cell, showing the cell in lighter grey tones against a darker, uniform background. At this magnification only clusters of gold labels are visible. (B) Image recorded at the position of the dashed rectangle in (A) at a higher magnification where labels become visible. (C) The gold labels are visible as individual particles. The inset shows the labels at the highest magnification in this imaging series. (D) Two types of beam damage occurred after the imaging series. A dark round shape is visible at the left and white shapes are visible at the top.

Mammalian COS7 cells were grown directly on the silicon nitride membranes of the microchips and incubated for 5 minutes with gold-tagged EGF (10 nm diameter gold nanoparticles). The incubation of the cells with the gold-tagged EGF for 5 minutes is expected to be sufficient for EGF binding, but not for complete receptor internalization [19]. The gold labels should thus be found preferentially at the cells' surface. **Figure 6** shows a series of images recorded at the edge of a COS7 cell. In **Figure 6**A, the contour of the cell is visible as light grey matter over a dark gray background at the top and at the right. Some internal structures of the cell can be observed as well. Individual gold labels cannot be distinguished at this magnification, but clusters of labels are visible as bright spots in the image. **Figure 6**B is an image recorded at a higher magnification at the position of the dashed rectangle in **Figure 6**A, showing several tens of labels. **Figure 6**C shows a second area in which the labels can be distinguished. After ligand-binding at the cell's surface the EGF receptor is know to internalize via the formation of endocytotic vesicles [19]. The incubation time used here is too short for the complete formation of such endocytotic vesicles. Several labels in close proximity are visible in the circled area. This shape could present the initial phase of vesicle formation. One set of labels is also shown at the highest resolution in the inset. The 20–80% edge width of line-scans over the two smallest nanoparticles in the middle was 3 nm, which is considered to be the resolution obtained on this sample. The full width at half maximum was 10 nm, which measures the size of the nanoparticles. The cluster of 7 nanoparticles (circled area) can also be recognized in **Figure 6**B. These results show that wet STEM imaging can be used to study the spatial distribution of activated receptors.

Radiation damage occurred after recording this series of images (**Figure 6**D). The whole series of images acquired in this area consisted of 6 images, recorded at magnifications of 3 k (**Figure 6**A), 5 k (not shown), 10 k (not shown), 10 k (**Figure 6**B), 40 k (**Figure 6**C), and 110 k (**Figure 6**C inset). Three types of radiation damage can be distinguished. At the left, a dark round shape is visible. In STEM imaging darkening means that less material resides in the beam path. The dark shape thus represents a void in the liquid, which could have been a bubble of gas. The formation of nano-bubbles is a known phenomenon from the imaging of frozen samples [20], [21]. At the upper side of the image a white shape is visible indicating a concentration of material, which could possibly have been caused by the build-up of carbon contamination. Contamination could have occurred both inside the liquid enclosure, as well as on the vacuum side of the silicon nitride window. Thirdly, we have measured the distance between gold nanoparticles in two clusters to evaluate structural damage to the cellular material where the labels were bound. The largest distance in the cluster of four labels, shown in the inset of **Figure 6**C, was measured to be 127±1 nm and this distance was the same for **Figure 6**B, C, and D. The largest distance between nanoparticles in the circled cluster was 92 nm for **Figure 6**C and equal to the distance determine from **Figure 6**B within the error, but changed to 101 nm in **Figure 6**D, indicating the occurrence of structural damage. Thus, repeated imaging of the same area is possible without structural damage, until a threshold electron dose has been achieved and different forms of radiation damage occur.

## Discussion

Our results demonstrate wet-STEM imaging of labeled bacterial- and eukaryotic cells with nanometer resolution. The STEM imaging method provides an order of magnitude higher spatial resolution than nanoscopy techniques [1], [2] for imaging times of several seconds. The sample preparation method is similar to methods used for fluorescent light microscopy on fixed cells labeled with quantum dots or other fluorescent nanoparticles. The micro-environmental chamber made of two silicon microchips can be assembled in a matter of minutes (drying took several hours, but a fast-drying glue can be used if needed). The advantages of our approach with respect to conventional biological electron microscopy on thin sections, e.g., using immunogold labeling [5] are, 1) the possibility of imaging whole cells and 2) the absence of sample preparation steps involving staining (e.g., with compounds containing osmium, or lead), drying, and slicing [22]. A further advantage is that the wet STEM system is rather simple. The entire system requires a STEM, which is already available on many TEMs, a standard specimen holder modified to contain a slot for the microchips (this is a minor modification), the silicon microchips, and a loading tool. Wet-STEM presents a simple alternative to cryo-EM for the case of biological experiments where distributions of labeled components are to be investigated and imaging of the full intracellular ultrastructure is not needed. The wet STEM system introduced here does not require dedicated equipment such as an *in situ* TEM, an environmental scanning electron microscope, or a specimen holder for liquid flow.

It is important to stress the difference between the information in wet-STEM images and in conventional TEM images. The images of wet-STEM reveal nanoscale information about the distribution of labeled components, but differ from standard EM images by the absence of high-resolution information of (stained) cellular components such as membranes and organelles. Conventional TEM provides contrast, e.g., on the whole ultrastructure of a cell in a thin section. Wet-STEM exhibits only moderate resolution and contrast on the cellular material. High-resolution is then obtained on the labels visible on a surrounding background of a much lower signal than in TEM. In other words, the reduced amount of information with respect to TEM lets us observe specific labels while being able to “see through” the cell. This effect has been demonstrated for (dry) thin sections by others [23]. The comparison between wet-STEM and TEM has its analogy in light microscopy, where fluorescently tagged proteins are imaged with fluorescent techniques, and unlabelled cellular structure is typically viewed with phase contrast techniques. Wet-STEM can thus be used to study (multiple) protein distributions with high resolution, but one has to preselect and label the proteins to be studied.

From the repeated imaging of the same specimen region at increasing magnifications it was found that the limit of radiation damage was reached at a magnification of 110 k (**Figure 6**C inset). The associated electron dose can be estimated from the irradiated specimen area of 1.0 µm^2^ (note that the area actually shown in the inset of **Figure 6**C is smaller), which gives an electron dose of 1⋅10^4^ electrons/nm^2^. The images recorded prior to the **Figure 6**C inset were recorded at lower magnifications and the total dose was only 10% of that of the **Figure 6**C inset. Here, we neglect that the STEM probe was smaller than the pixel size and that the radiation dose may have been locally higher at the focus. The dose of 1⋅10^4^ electrons/nm^2^ is just above the limit for the imaging of frozen biological material in tilt-series TEM [24]. The beam damage limit occurring during wet-STEM imaging of fixed cells in liquid thus appears to be comparable with that for TEM imaging of frozen samples. Frozen samples are expected to be more stable under the electron beam than samples at room temperature, because after the breaking of atomic bonds by radiation induced excited states, the atoms would not diffuse to other locations. This type of radiation damage only becomes visible after thawing the sample. On the other hand, a liquid would allow charge carriers and radicals to diffuse and thus prevent local damaging of the specimen. The removal of charge carriers and radicals outside a cell is expected to be enhanced by continuous liquid flow [12]. Another radiation damage effect, the formation of gas bubbles known from cryo-TEM, was observed for wet-STEM as well.

Wet-STEM using our micro-environmental chamber provides at least a factor of 5 higher resolution on gold nanoparticles used as labels on cells than obtained with a liquid capsule for SEM imaging with the backscatter detector [10] and similar commercially available systems. A further advantage of the wet-STEM over SEM-based approaches is that STEM is not a surface technique. The SEM obtains high resolution only from the very top layer (few tens of nanometers) of the sample, whereas we have already demonstrated nanometer resolution on gold nanoparticles at a depth of 1.3 µm in the liquid [12]. In another wet-STEM imaging approach using environmental SEM equipment [25] the obtained resolution for the imaging of whole cells was limited due to electron-sample interactions. For the 30 keV electron beam in SEM, the mean free path length for elastic scattering [20] into a STEM detector with a semi-angle of 70 mrad, is only 0.4 µm. In contrast, the higher beam energy of the 200 keV STEM used for our imaging method, results in an increase of the corresponding mean free path length to 11 µm [12], and nanoscale imaging can be obtained on whole cells. Due to the sensitivity of STEM imaging on the atomic number, the resolution obtained on labels embedded in thick regions of cellular material and liquid is much higher than achievable with a TEM using liquid-[11] and liquid-vapor enclosures [26], [27]. The contrast mechanism of TEM, with its sensitivity to materials of a low atomic number, prevents the imaging of thick specimen. The TEM has traditionally been used to image thin (smaller than 0.5 µm) samples of biological material and the imaging of whole eukaryotic cells is not possible with a resolution better than that of light microscopy.

The wet environment maintains fixed cells in a liquid state, which preserves their structure as concluded for environmental SEM [13], and avoids dehydration and/or slicing artifacts as observed in conventional biological electron microscopy. Wet-STEM can be applied to a range of biological experiments involving nanoscale labels/materials [28]. New nanoparticle based reagents for molecular imaging [29] can be tested at the cellular level on specificity. Wet-STEM could be especially helpful when nanoparticles are used as labels. In cell biology receptor-function can be studied by incubating cells for various time intervals with a ligand. The cellular response to incubation can then be elucidated by comparing patterns and positions of the labels, for example, to track internalization of the EGF receptor via endosomes [12], [19]. The system presented here may also be helpful to investigate binding events happening in and on bacteria, for example, toxic effects associated with nanoparticles, or to develop specific bacterial tags [30].

In conclusion, the wet-STEM method presented here is capable of imaging nanoparticles in/on whole fixed cells in liquid state. The resolution obtained on gold labels on COS7 cells was 3 nm. We expect that the capability to image whole cells, the compatibility of the sample preparation with light microscopy, and the inexpensive equipment will spur the nanoscale imaging of protein distributions in whole cells in biological electron microscopy laboratories.
